# Transcriptomic, Functional, and Network Analyses Reveal Novel Genes Involved in the Interaction Between *Caenorhabditis elegans* and *Stenotrophomonas maltophilia*

**DOI:** 10.3389/fcimb.2018.00266

**Published:** 2018-08-20

**Authors:** Corin V. White, Michael A. Herman

**Affiliations:** Ecological Genomics Institute, Division of Biology, Kansas State University, Manhattan, KS, United States

**Keywords:** *Caenorhabditis elegans*, *Stenotrophomonas maltophilia*, WormNet, network, expression, innate immunity

## Abstract

The bacterivorous nematode *Caenorhabditis elegans* is an excellent model for the study of innate immune responses to a variety of bacterial pathogens, including the emerging nosocomial bacterial pathogen *Stenotrophomonas maltophilia*. The study of this interaction has ecological and medical relevance as *S. maltophilia* is found in association with *C. elegans* and other nematodes in the wild and is an emerging opportunistic bacterial pathogen. We identified 393 genes that were differentially expressed when exposed to virulent and avirulent strains of *S. maltophilia* and an avirulent strain of *E. coli*. We then used a probabilistic functional gene network model (WormNet) to determine that 118 of the 393 differentially expressed genes formed an interacting network and identified a set of highly connected genes with eight or more predicted interactions. We hypothesized that these highly connected genes might play an important role in the defense against *S. maltophila* and found that mutations of six of seven highly connected genes have a significant effect on nematode survival in response to these bacteria. Of these genes, C48B4.1, *mpk-2, cpr-4, clec-67*, and *lys-6* are needed for combating the virulent *S. maltophilia* JCMS strain, while *dod-22* was solely involved in response to the avirulent *S. maltophilia* K279a strain. We further found that *dod-22* and *clec-67* were up regulated in response to JCMS vs. K279a, while C48B4.1, *mpk-2, cpr-4*, and *lys-6* were down regulated. Only *dod-22* had a documented role in innate immunity, which demonstrates the merit of our approach in the identification of novel genes that are involved in combating *S. maltophilia* infection.

## Introduction

*Stenotrophomonas maltophilia* is an emerging nosocomial and opportunistic bacterial pathogen associated with mortality rates ranging from14 to 69% in patients with bacteremia (Brooke, 2012). Over the last decade, *S. maltophilia* infections have increased in prevalence among the general population (Chang et al., 2015). These infections can be acquired both in the community (Falagas et al., 2009; Chang et al., 2014) or associated with health care (Garazi et al., 2012). Like other nosocomial pathogens, *S. maltophilia* is associated with respiratory tract, soft tissue, and skin infections and can exacerbate the effects of other diseases and disorders such as chronic obstructive pulmonary disease (Denton and Kerr, 1998; Brooke, 2012). *S. maltophilia* is also multidrug resistant and antibiotic resistance genes have been found in clinical and environmental strains (Sánchez, 2015). Environmental isolates are found in bottled water, plant rhizospheres, a variety of soil types, and water sources (Denton and Kerr, 1998; reviewed in Brooke, 2012). Thus, *S. maltophilia* is found ubiqutiously, and the presence of intrinsic antibiotic resistance in combination with the recent increase in the prevelance of infections renders the study of its interaction with hosts a major priority.

Despite its biomedical significance, there is still much to be learned about how *S. maltophilia* influences host immune response. The nematode *Caenorhabditis elegans* has been shown to be an effective model for the study of host-pathogen interactions (reviewed in Irazoqui et al., 2010a; Marsh and May, 2012; Kim, 2013). We and others have discovered a pathogenic interaction between *C. elegans* and *S. maltophilia* (Fouhy et al., 2007; Thomas et al., 2013; Jankiewicz et al., 2016; White et al., 2016). Additionally, *Stenotrophomonas* and other Proteobacteria, dominate the *C. elegans* microbiome (Dirksen et al., 2016). Thus, the study of this interaction has both ecological and evolutionary significance. Previously, we found that while the *C. elegans* response to *S. maltophilia* has some common elements, aspects of the interaction are specific to individual *S. maltophilia* strains (White et al., 2016). Therefore, a major aim of this study was to identify genes that underlie strain specific nematode innate immune response. To do so, we used a transcriptomic approach to identify genes that were differentially expressed in the *C. elegans* response to pathogenic and non-pathogenic *S. maltophilia*.

Previous studies involving the transcriptomic response of *C. elegans* to a variety of bacteria provide evidence for a nematode innate immune response that involves both common and unique molecular mechanisms. The genes identified in these studies typically share common molecular functions, including those involved in ion channel activity, sugar and lipid binding, proteolysis, and lysozyme activity (Troemel et al., 2006; Wong et al., 2007; Coolon et al., 2009; Irazoqui et al., 2010b; Visvikis et al., 2014). This transcriptional response is shared between, and is specific to, different bacterial pathogens. For example, genes involved in stress response, insulin signaling and cell death are commonly differently expressed in *C. elegans* exposed to *Enterococcus faecalis, Erwinia carotovora*, and *Photorhabdus luminescens*, while only *E. faecalis* exposure is associated with a down-regulation of hormone receptors (Wong et al., 2007). Similarly, the *C. elegans* transcriptional response to *S. aureus, Pseudomonas aeruginosa*, and *Microbacterium nematophilum* included both common and unique genes (Irazoqui et al., 2010b). For example, some of the overlapping induced genes had functions in intracellular detoxification and iron sequestration, while genes that were expressed on *Staphylococcus aureus* and *P. aeruginosa* are associated with the expression of transferases, proteases and lipases (Irazoqui et al., 2010b). Thus, we also hypothesized that profiling *C. elegans* gene expression in response to *E. coli* and *S. maltophilia* isolates would further elucidate such common and unique mechanisms.

A common issue for the analysis of genes identified by transcriptional profiling is how to prioritize candidates for further study. The use of gene network models such as WormNet to prioritize genes for validation has been shown to be useful in the study of a variety of responses in nematodes, plants and humans (Lee et al., 2008, 2010a,b; Huttenhower et al., 2009; Zugasti et al., 2016). Specifically, genes that are connected in WormNet are likely to have similar loss of function phenotypes and the authors observed that gene connectivity was correlated with the frequency of non-viable RNA interference (RNAi) phenotypes (Lee et al., 2008). A similar approach using connectivity to predict function, helped to identify human proteins involved in macroautophagy through the query of a functional map with known autophagy proteins (Huttenhower et al., 2009). Thus, probabilistic network connections have proven predictive power and can aid in the identification of genes associated with similar traits and we have used this approach here to identify genes with functional significance for the *C. elegans—S. maltophilia* interaction. Genes with a greater number of connections within the probabilistic network model (generated via WormNet) were termed “hubs” (Özgür et al., 2010; Cho et al., 2014). We hypothesized that these hubs were critical to the nematode bacterial response because they were differentially expressed and central in the network. We then used mutations in several of these genes to show that all but one hub were in fact required on at least one of the *S. maltophilia* isolates tested. Several additional differentially expressed genes that are associated with enriched terms also had significant phenotypes. Most of the genes that had survival phenotypes have no documented role in *C. elegans* innate immune response, which supports the merit of using this approach. Our data reveals that the *C. elegans* innate immune response is specific to bacterial pathogenicity rather than taxonomic classification. This response includes functions and processes that have been shown to be involved in other nematode-bacterial pathogen interactions.

## Materials and methods

### Nematode strains

The following *C. elegans* strains containing the designated alleles were obtained from the Caenorhabditis Genetics Center (CGC): *N2*, LG I: *kcnl-2(ok2818)*, LG II: *acr-7(tm863), mpk-2(ok219)*, LG III: C48B4.1*(ok2619), numr-1(ok2239)*, LG IV: *dod-22(ok1918), clec-67(ok2770), lys-6(ok2075), tctn-1 (ok3021)*, LG V: *cpr-4(ok3413), gcy-14(pe1102), srw-145(ok495)* LG X: *acs-17(ok1562), lgc-11 (tm627)*. Strains containing *mpk-2*, C48B4.1*, dod-22, clec-67, lys-6*, and *cpr-4* were outcrossed four times and *acs-17* was outcrossed twice. Following each outcross, segregates were screened via PCR to obtain nematodes that were homozygous for the deletion allele at the desired locus. The inner and outer primer sequences used for screening are available from the CGC and WormBase. *N2* was used as the wild-type strain for outcrossing and survival analyses. This strain is kept frozen at −80°C and thawed yearly for experimentation.

### Bacterial strains and growth

*S. maltophilia* JCMS was isolated by our laboratory from a culture of *Mesorhabditis* sp. found in soils from Konza Prairie, near Manhattan, KS as previously described (White et al., 2016). *E. coli* OP50 was obtained from the CGC and *S. maltophilia* K279a from R. Ryan (University College Cork). All bacterial strains were frozen at −80°C upon retrieval and were thawed regularly for use in our experiments. *S. maltophilia* strains are naturally Ampicillin resistant and were streaked for colony isolation from frozen stock on Luria Broth (LB) agar containing 100 μg/mL Ampicillin to selectively prevent growth of other bacterial contaminants. *E. coli* OP50 was streaked on LB agar for colony isolation. For each bacterial strain, liquid LB was inoculated and shaken overnight at 32°C. Bacterial lawns used for survival were seeded on nematode growth medium (NGM) with bacterial culture at log/lag phase and grown overnight at room temperature.

### Nematode survival assays

Nematodes were reared and synchronized as L4s at 20°C on *E. coli* OP50 lawns. For survival analysis, 10–15 L4s were picked onto three to six replicate lawns of the treatment or control bacteria and maintained at 25°C. The number of surviving nematodes was recorded daily and death was determined by lack of motion in response to prodding with a platinum wire pick. Nematodes were picked to new bacterial lawns for the first 5–6 days after the start of the experiment to separate them from their progeny. Dead nematodes were removed upon discovery. Sample sizes (*N* = number of nematodes) varied due to the removal of replicates because of contamination and the removal of specimens that died via means other than the specified bacterial treatment, such as desiccation that occurs when nematodes leave the bacterial lawn and die at the plate edge. The presence of contamination was infrequent and was determined by observing bacterial lawn morphology. Kaplan-Meier estimates of survival over time and survival curve statistics using Cox proportional hazard tests were performed in R (Vienne, Austria: R Foundation for Statistical Computing). Survival curves can be statistically compared using the log-rank and Cox proportion hazard tests. Cox proportion hazard models were used to test the effect of independent variables such as genotype and bacteria on the hazard, a dependent variable defined as the probability of dying at a given time (Goel et al., 2010). Models were evaluated by testing for a non-zero slope and visualizing the Schoenfeld residuals. A non-zero slope is an indication of proportional hazard assumption violation and models were fit to the data aiming to meet that assumption.

### Bulk nematode RNA extraction

Synchronized wild-type nematodes were reared at 20°C on *E. coli* OP50 from egg to larval stage 4 (L4). L4s were then washed off the rearing plates with M9 buffer and placed onto several lawns of *S. maltophilia* JCMS, K279a or OP50. After 24 h of feeding on the treatment bacteria at 25°C, young adult nematodes were collected in M9 buffer and lysed in TRIzol® reagent (Life Technologies). Only non-contaminated, un-starved nematode populations were used. This bulk extraction was considered one biological replicate and was repeated four times for each bacterial treatment for the microarray experiment and three times for RT qPCR analyses. RNA extraction and DNAse treatment were performed using the PureLink RNA Mini Kit (Invitrogen) and on-column PureLink® DNase Treatment (Invitrogen). RNA quality was checked by visualizing 28S and 18S rRNA bands using gel electrophoresis and checking 260/280 and 260/230 absorbance ratios using a NanoDrop™ 2000 Spectrophotometer. RNA extraction was performed similarly for downstream applications.

### Reverse transcription quantitative polymerase chain reaction (RT qPCR)

**Figure 1:**
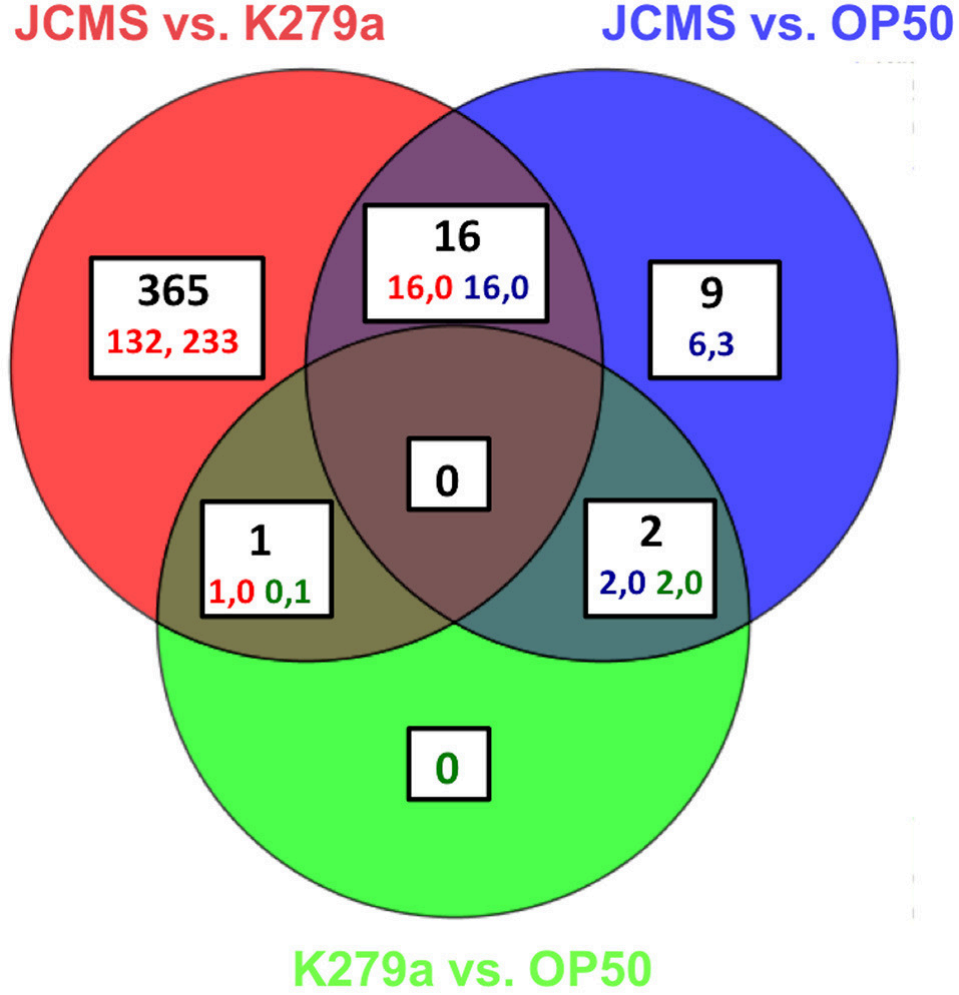
All significantly differentially expressed genes for wild-type nematodes fed *E. coli* OP50, *S. maltophilia* JCMS or K279a. Differential expression was determined on all pairwise comparisons of *C. elegans* adults exposed to *S. maltophilia* JCMS, *S. maltophilia* K279a and *E. coli* OP50. OP50 or K279a was the baseline treatment for each comparison. Statistical significance was determined using a moderated *T*-test and a Benjamini–Hochberg multiple testing correction with a 1.5-fold change cut off. The regulation and corrected p value for each gene is listed in Table S3. A gene was considered significantly differentially expressed if the corrected *p* < 0.05. 393 genes were significantly differentially expressed. The regulation (up, down) of each comparison is indicated for each category. The colored numbers indicate the number of comparisons, which are double for the three overlapping categories.

Intact RNA was used for cDNA synthesis using a SuperScript® VILO cDNA Synthesis Kit (Invitrogen). RT qPCR was performed using the CFX96 Touch™ Real-Time PCR Detection System (BIO RAD). Each amplification reaction was performed in triplicate and three biological replicates of pooled bulk nematode RNA extraction were performed for each bacterial and nematode combination. We chose genes that had the potential to validate the pairwise comparisons between bacterial treatments depicted in Figure 1. These genes were chosen solely for the validation of the observed microarray expression pattern. Each gene was chosen based on its occurrence in one or two bacterial treatment comparisons, a minimum 2.5-fold change value (see Microarray analysis) and ease of primer efficiency optimization. The reference gene *csq-1* was used because it was not significantly differentially expressed between the bacterial treatments in this study. *csq-1* has also been shown to be a reliable reference gene (Wu et al., 2014) and primers for *csq-1* maintained a high efficiency across the different RT qPCR reactions required to amplify the target genes. The efficiency of each primer pair was determined using a standard curve on each biological replicate of cDNA collected on JCMS, K279a, and OP50. The efficiencies of the target and *csq-1* primers were approximately equal (Applied Biosystems) and thus were assumed to be 100% during Δ*C*_T_ quantification. Primer sequences for *csq-1*, F53B2.8, W03F9.4, *ilys-3* (C45G7.3), and F08G2.5 were designed using Geneious ®6.1.8 and checked for specificity using NCBI BLAST (nucleotide collection nr/nt database) and are provided in Table S1. Differential expression was determined by comparing the 2^−ΔCT^ values for biological replicates of the target gene on *S. maltophilia* JCMS or K279a and *E. coli* OP50 in wild-type nematodes (Schmittgen and Livak, 2008). Statistical significance was determined with a Student's *t*-test assuming equal variance.

### Microarray target preparation for hybridization

cDNA was synthesized using the SMARTer PCR cDNA synthesis kit (Clontech), amplified and optimized using the Advantage 2 PCR Kit (Clontech). Two hundred and fifty nanograms of total RNA was reverse transcribed using a modified oligo dT primer and SMARTScribe reverse transcriptase, followed by second strand cDNA synthesis. PCR cycling parameters for second strand synthesis were optimized to ensure that the generated dsDNA remained in the exponential phase of amplification. The phase of amplification was evaluated by observing the double stranded cDNA smear of each sample using gel electrophoresis. Four biological replicates of pooled bulk nematode RNA extractions were used for each bacterial nematode combination. Double-stranded cDNA was purified using the PureLink™ Quick PCR Purification Kit (Invitrogen) and ethanol precipitation. DNA quality was checked by observing the 260/280 and 260/230 absorbance ratios using a NanoDrop™ 2000 Spectrophotometer. Purified cDNA was sent to NimbleGen Gene Expression Services (Roche NimbleGen Inc.) for fragmentation and hybridization on one *C. elegans* Gene Expression 12 × 135K chip containing 12 single color arrays (one array per biological replicate). Each array contained a total of 136,883 probes (5 to 6 probes per gene) representing the entire *C. elegans* transcriptome (23,196 genes).

### Microarray analysis

Summarized and baseline transformed files (NimbleGen) from all 12 arrays (4 per bacterial treatment) were uploaded into GeneSpring 12 (Agilent Technologies) and quantile normalized. Quality control results from principal components analysis, a correlation table and correlation coefficients were used to evaluate the similarity among biological replicates within each treatment (Table S2 and Figure S1). One biological replicate for nematodes exposed to *E. coli* OP50 was determined to be an outlier and removed from the analysis of differential expression. Following outlier removal, the biological replicates were grouped by treatment. Bacterial treatments were then compared within GeneSpring using a moderated *T-*test (Baldi and Long, 2001) and a Benjamini-Hochberg multiple testing correction with a 1.5-fold change cut off. Genes with a 1.5-fold or greater change and a Benjamini–Hochberg corrected *p* < 0.05 were considered significant and are listed with their associated bacterial treatment comparison in Table S3. Microarray data was deposited in the Gene Expression Omnibus (GEO) archive with accession number GSE107272.

### Gene ontology and enrichment analysis

Significant enrichment of gene ontology (GO) terms was determined using DAVID Bioinformatics Resources 6.8, NIAID/NIH. GO enrichment analyses were performed on separate lists of 157 up-regulated and 237 down-regulated genes. These gene lists represent all differentially expressed genes among all bacterial treatments. We chose not to separate by comparison since 382 genes were differentially expressed between JCMS and K279a, which would leave a small and statistically unfavorable sample size for the other comparisons. ZK105.5 was significantly up regulated on *S. maltophilia* JCMS (2.03-fold, corrected *p* = 0.041) and down regulated on K279a (3.23-fold, corrected *p* = 0.047) as compared to expression on *E. coli* OP50 and was included in both lists used for enrichment analyses. The Functional Annotation Tool in DAVID was queried with separate lists of Wormbase ID numbers with the entire *C. elegans* transcriptome set as background. Nine up regulated genes and one down regulated gene were not mapped with annotations in DAVID and were excluded from the analysis (Table S4). Databases containing all biological process (BP), molecular function (MF), and cellular component (CC) GO terms were analyzed with and visualized using the Functional Annotation Chart view. Each GO term had an associated category, count of genes, percentage of genes, *p*-value (modified Fisher's exact test) and multiple testing corrected p values (Benjamini–Hochberg). GO terms with significant corrected *p*-values (*p* < 0.05) were considered enriched and only the most specific GO terms with unique gene sets were included (Table 1). Enriched gene lists for each term are included in Table S4.

**Table 1:** GO analysis of up and down regulated differentially expressed genes in response to *S. maltophilia* JCMS, K279a or *E. coli* OP50.

	**Term**	**Count**	**%**	**Fold enrichment**	**Benjamini-Hochberg**
**UP REGULATED GENES**					
BP	Response to stimulus	33	22.3	1.8	7.55E-03
	Response to stress	28	18.9	4.7	1.16E-10
	Innate immune response	26	17.6	15.0	7.76E-21
	Defense response to Gram-negative bacterium	6	4.1	13.2	4.85E-03
CC	Membrane raft	7	4.7	19.6	7.97E-05
**DOWN REGULATED GENES**					
BP	Single-organism metabolic process	39	16.5	1.84	1.20E-02
	Oxidation-reduction process	24	10.2	3.14	3.86E-04
	Flavonoid glucuronidation	7	3.0	7.69	1.42E-02
	Single-organism localization	35	14.8	1.79	3.05E-02
	Transmembrane transport	30	12.7	2.69	8.82E-04
	Ion transport	27	11.4	2.89	4.82E-04
	Ion transmembrane transport	22	9.3	3.37	3.10E-04
	Cation transport	17	7.2	3.07	1.00E-02
	Cation transmembrane transport	34	14.4	1.84	2.44E-02
	Inorganic ion transmembrane transport	17	7.2	3.77	1.14E-03
	Metal ion transport	15	6.4	4.55	6.54E-04
	Monovalent inorganic cation transport	12	5.1	3.90	1.35E-02
	Inorganic cation transmembrane transport	12	5.1	3.39	3.27E-02
	Potassium ion transmembrane transport	8	3.4	7.26	1.03E-02
MF	Transporter activity	31	13.1	2.18	9.05E-04
	Transmembrane transporter activity	29	12.3	2.42	3.21E-04
	Substrate-specific transmembrane transporter activity	24	10.2	2.49	1.18E-03
	Ion transmembrane transporter activity	22	9.32	2.76	7.36E-04
	Channel activity	23	9.75	4.34	8.84E-07
	Substrate-specific channel activity	22	9.32	4.59	1.19E-06
	Cation transmembrane transporter activity	15	6.36	2.55	2.01E-02
	Ion channel activity	21	8.90	4.55	1.35E-06
	Cation channel activity	14	5.93	4.77	1.90E-04
	Metal ion transmembrane transporter activity	12	5.08	3.41	8.85E-03
	Monovalent inorganic cation transmembrane transporter activity	11	4.66	3.35	1.58E-02
	Potassium ion transmembrane transporter activity	8	3.39	6.06	5.04E-03
	Oxidoreductase activity	24	10.2	2.88	1.83E-04
	Monooxygenase activity	14	5.93	9.64	4.19E-07
	Oxidoreductase activity, acting on paired donors, with incorporation or reduction of molecular oxygen	13	5.51	6.77	1.79E-05
	Steroid hydroxylase activity	5	2.12	8.97	2.02E-02
	Heme binding	13	5.51	5.76	8.69E-05
	Transmembrane receptor activity	28	11.9	2.02	6.82E-03
	Gated channel activity	13	5.51	4.84	3.29E-04
	Ligand-gated ion channel activity	9	3.81	4.69	8.33E-03
	Extracellular ligand-gated ion channel activity	8	3.39	4.74	1.48E-02
	Excitatory extracellular ligand-gated ion channel activity	6	2.54	5.38	4.11E-02
	Extracellular-glutamate-gated ion channel activity	4	1.69	10.91	4.44E-02
	Glucuronosyltransferase activity	7	2.97	6.45	8.79E-03
	Transmembrane signaling receptor activity	24	10.2	1.87	3.43E-02
	Iron ion binding	14	5.93	7.89	1.27E-06
CC	Membrane	104	44.1	1.35	1.51E-05
	Membrane part	102	43.2	1.39	6.28E-06
	Cell periphery	34	14.4	2.04	1.45E-03
	Plasma membrane part	23	9.7	2.09	1.37E-02
	Integral component of membrane	101	42.8	1.46	8.27E-07
	Synaptic membrane	5	2.1	7.47	4.68E-02
	Integral component of plasma membrane	20	8.5	2.40	9.82E-03
	Ion channel complex	8	3.4	5.60	1.11E-02

*148 up and 236 down regulated genes were analyzed with the functional annotation tool using databases containing all biological process (BP), molecular function (MF) and cellular component (CC) GO terms and visualized using the Functional Annotation Chart view in DAVID Bioinformatics Resources 6.8. Significant Fold-enrichment and Benjamini-Hochberg corrected p-values (implemented by DAVID to control for multiple testing) are reported. Only GO terms with a corrected p < 0.05 are shown and additional significantly differentially expressed isoforms of the same gene were not included in the analysis. Gene lists for each term are included in Table S4. Indention indicates relative parent-child term relationships with parent terms listed first and left most in the “Term” column. Parent terms such as “response to stress” are more generalized and encompass child terms with more specificity such as “defense response to Gram-negative bacterium.” Note that the degree of indention does not reflect absolute GO term levels between groups of terms and a term may have more than one parent term. The “Count” column is number of unique genes corresponding to each term. Percent is that of the total number analyzed*.

### Tissue enrichment analysis

Significant tissue/anatomy term enrichment analysis (TEA) was also performed on separate lists of up and downregulated genes, as described above, using Wormbase Version WS260 and the Wormbase ID numbers of the differentially expressed genes (Angeles-Albores et al., 2016). One hundred and thirty-six of one hundred fifty-seven up and 204 of 237 down regulated genes had annotated data and were included in statistical analysis (Table S4). Significant fold change enrichment and a standard FDR (false discovery rate) correction using a Benjamini-Hochberg algorithm were reported in TEA output as q values. Only anatomy terms with a *q* < 0.05 are reported in Table 2.

**Table 2:** Tissue enrichment analysis of up and down regulated genes.

**Tissue**	**Count**	**%**	**Fold enrichment**	**Benjamini–Hochberg**
**UP REGULATED GENES**				
Intestine	77	56.6	2.5	<1.0E-06
Muscular system	44	32.4	1.6	2.80E-02
Outer labial sensillum	31	22.8	2	0.016
PVD	31	22.8	2	0.016
Striated muscle	18	13.2	2.3	0.023
AVA	10	7.4	3.2	0.023
**DOWN REGULATED GENES**				
Intestine	92	45.1	1.4	0.027
Amphid sensillum	67	32.8	1.4	0.041
Lateral ganglion	48	23.5	1.9	0.00081
ASE	40	19.6	1.9	0.0032
AWB	20	9.8	2	0.029
Inner labial sensillum	17	8.3	3.2	0.00081
IL2 neuron	16	7.8	3.7	0.00058
Ray	16	7.8	3.3	0.00081
Hook sensillum	15	7.4	3.7	0.00058
Amphid sheath cell	15	7.4	2.1	0.055
CEM	14	6.9	3.5	0.00081
AVE	13	6.4	2.5	0.025

*136 up and 204 down regulated genes were queried for Tissue Enrichment Analysis (TEA) at Wormbase (Version WS258). The number of genes associated with each anatomy term/tissue (count), fold change enrichment and a standard FDR correction (Benjamini-Hochberg corrected p values < 0.05) are reported. Percentage of the analyzed genes was calculated using the associated count data. Anatomy term definitions are as follows: PVD, Posterior interneurons; AVA and AVE, Interneurons in the lateral ganglia; ASE and AWB, Ciliated neurons that are part of the amphid sensilla; IL2 neuron, Ciliated neurons of the inner labial sensilla, Hook sensillum, and Ray, male sensory organs; CEM, Neuron class of four male-specific neurons*.

## Results

### *C. elegans* gene expression profiles are driven by bacterial pathogenicity

Previous studies on the interaction of *C. elegans* with pathogenic bacteria suggested that the accumulation of living bacteria in the intestine plays a large role in nematode pathogenesis (Garsin et al., 2001; Sifri et al., 2005). Furthermore, we previously observed that the pathogenic *S. maltophilia* isolate JCMS causes a significantly higher bacterial load 24 h post exposure as compared to other, less virulent *S. maltophilia* strains and the standard laboratory food *E. coli* OP50 (White et al., 2016). There is also a known decline in immune response with aging (Youngman et al., 2011) and an increase in putative pathogen recognition genes in *C. elegans* at 8 h in response to the bacterial pathogen *S. aureus* (Visvikis et al., 2014). Thus, we reasoned that 24 h of exposure represented an intermediate time point between pathogen detection and a gene signature that reflects the natural process of immune deterioration. To gain insight on how *C. elegans* combats exposure to JCMS, we conducted a transcriptomic study in which wild-type nematodes were exposed to *S. maltophilia* JCMS, *S. maltophilia* K279a and *E. coli* OP50 for 24 h. Gene expression was determined using microarrays for pairwise comparisons of all bacterial treatments. Four hundred fifty-four significantly differentially expressed transcripts representing 393 unique genes were identified (Figure 1, Table S3). Three hundred eighty-two of these genes were identified in comparisons of the two *S. maltophilia* strains, K279a and JCMS. Of these, 149 were up-regulated on JCMS and 233 genes were down-regulated. Twenty-seven genes were differentially expressed between JCMS and OP50 (24 up-regulated and 3 down-regulated) and 3 (2 up regulated and 1 down-regulated) between K279a and OP50 (Figure 1, Table S3. The small number of differentially expressed genes identified between *E. coli* OP50 and each of the *S. maltophilia* strains was surprising as the strains belong to different orders of gammaproteobacteria and display different levels of virulence to *C. elegans*. However, we have observed K279a to be less virulent than OP50 and neither caused a substantial bacterial load within the nematode (White et al., 2016). Thus, the majority of the observed transcriptional response appeared to be caused by differences in bacterial pathogenicity rather than taxonomy.

### Validation of expression profiles

**Figure 2:**
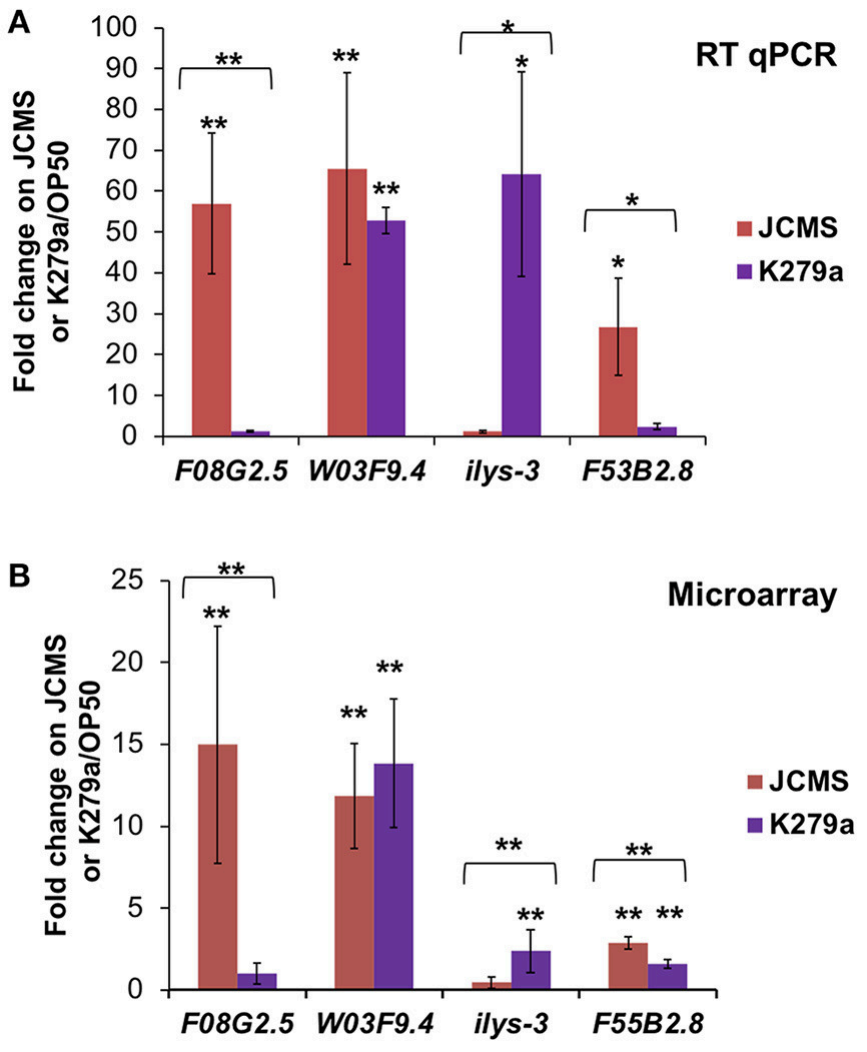
RT qPCR of several significantly differentially expressed genes validates the microarray dataset. Expression of F08G2.5, W03F9.4, *ilys-3*, and F53B2.8 in wild-type nematodes exposed to *S. maltophilia* JCMS (red) or K279a (purple). **(A)** RT qPCR **(B)** Microarray. Fold change is shown in reference to expression on *E. coli* OP50. Statistical significance (*p* < 0.05^**^ or 0.1^*^) was determined with a Student's *t*-test assuming equal variance. Asterisk(s) above the error bars: expression on JCMS or K279a was significantly different from on OP50. Asterisk(s) above a bracket: expression level of nematodes exposed to JCMS was significantly different from those exposed to K279a. The comparisons validated from the microarray experiment (Figure 1, Table S3) are as follows: F08G2.5 up-regulated on JCMS vs. K279a, *ilys-3* down-regulated on JCMS vs. K279a, F53B2.8 up-regulated on JCMS vs. K279a and OP50 and, W03F9.4 down-regulated on OP50 vs. K279a and JCMS.

Although we identified many differentially expressed genes between JCMS and K279a, the small number of genes identified in the JCMS vs. OP50 comparison was surprising. To further investigate the distribution of differentially expressed genes observed in each category, we validated several genes that were identified in one or two bacterial treatment comparisons. The genes chosen for validation occurred in at least one bacterial treatment comparison, had a minimum 2.5-fold change value and were amplified by primers that achieved consistently high efficiency across reactions. The genes and comparisons were: F08G2.5 up-regulated on JCMS vs. K279a, *ilys-3* down-regulated on JCMS vs. K279a, F20G2.5 up-regulated on JCMS vs. OP50, F53B2.8 up-regulated on JCMS vs. K279a and JCMS vs. OP50, and W03F9.4 up-regulated on K279a vs. OP50 and JCMS vs. OP50 (Table S3). The significance and regulation pattern of all genes except F20G2.5 were validated using RT qPCR (Figure 2). While we observed that F20G2.5 was up-regulated in response to JCMS vs. OP50 (data not shown), most of the Cq-values in response to OP50 were outside the range of detection and this gene was not included in Figure 2. F08G2.5 and *ilys-3* were also significantly differentially expressed between OP50 as compared to JCMS or K279a, which was detected using the microarray, but was not statistically significant. Thus, most of the expression differences for the selected genes were authenticated, suggesting that the overall expression pattern was correct.

### Gene ontogeny enrichment analyses

As an initial step to characterize the list of differentially expressed genes, we performed GO term enrichment analysis using DAVID (Huang et al., 2008, 2009). Of the 393 differentially expressed genes, 97.5% were mapped to annotations in DAVID and were used in enrichment analysis (Table S4). Of the annotated up regulated genes, 22.3, 18.9, and 4.7% were associated with biological process (BP) terms response to stimulus or stress and/or cellular component term membrane raft (Table 1). More specifically, 17.6 and 4.1% are involved in innate immunity and/or defense response to Gram-negative bacterium. These enriched terms were expected since most of these genes are differentially expressed in response to virulent vs. avirulent bacteria (Figure 1 and Table S3). For the down regulated genes, membrane (44.1%), membrane part (43.2%), and integral component of membrane (42.8%) terms were associated with the highest percentage of genes (Table 1). These cellular component terms correlated well with some biological process (ion, cation, inorganic cation, inorganic ion, and/or potassium ion transmembrane transport) and molecular function (ion, cation, and/or metal ion transmembrane transporter, receptor and/or signaling receptor activity) terms. Taken together, these results suggest that in response to *S. maltophilia* JCMS, *C. elegans* down regulates genes involved in membrane transport and oxidation-reduction and, up regulates genes that have a role in interacting with bacteria.

We also performed tissue enrichment analysis (TEA) using the TEA tool available at Wormbase (Table 2). 86.5% of the differentially expressed genes had the annotations necessary for statistical analysis. 56.6% of the up regulated genes and 45.1% of the down regulated genes are localized to the intestine which is the tissue where dysbiosis between bacterial prey and nematode predator is likely to occur first. The muscular system, outer labial sensillum, striated muscle as well as the AVA and PVD interneurons that run the length of the body also have genes with increased expression. On the other hand, the down regulated genes are localized to the amphid sensory structure, including the ASE and AWB sensory neurons as well as the amphid sheath cell. Down regulated genes are also localized to the inner labial and hook sensilla, including the IL2 sensory neurons, lateral ganglion, ray sensillum and the head sensory CEM cells that secrete pheromones as well as the AVE ventral cord interneurons involved in locomotion. Thus, these data reveal a sensory tissue specific decrease in expression and an increase of expression in tissues involved in locomotion.

### The nematode bacterial response gene network

**Figure 3:**
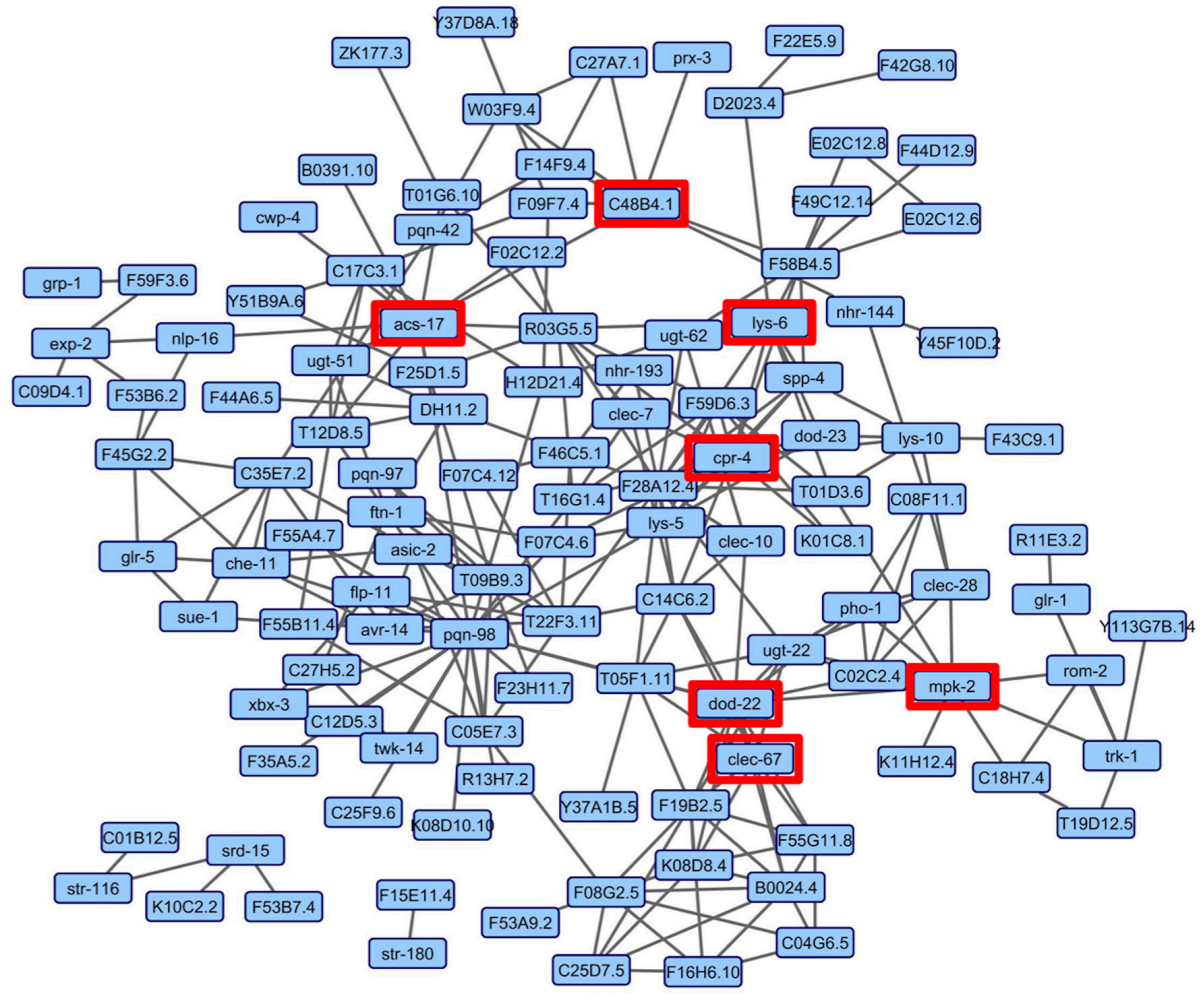
WormNet v2 network of differentially expressed genes on *S. maltophilia* JCMS, K279a or *E. coli* OP50. Probabilistic interactions were generated in WormNet v2 (Lee et al., 2008, 2010b) and the image was generated in Cytoscape 3.6.1. The gene network model includes 118 of 393 unique differentially expressed genes (Tables S3, S5). The area under the receiver operating characteristic (ROC) curve (AUC) value for the network was 0.896. Each blue box in the network represents a gene and the gray lines connecting genes are putative functional interactions. The hub genes chosen for validation are boxed in red.

Although the enrichment analyses allowed the sorting of genes by tissue and into functional categories, we sought a more impartial method to nominate genes for functional validation. We reasoned that such a method would be more likely to identify novel genes involved in the *C. elegans*-*S. maltophilia* response. WormNet v2 is a probabilistic functional gene network tool that employs a modified Bayesian integration of data from several different sources to measure the probability (log-likelihood score) of protein coding gene interactions (Lee et al., 2008). WormNet v2 contains 999,367 functional linkages between 15,139 genes which represents 75.4% coverage of the *C. elegans* protein-coding loci (Lee et al., 2010b). We queried WormNet v2 with all 393 differentially expressed genes and found 118 to have predicted interactions (Figure 3, Table S5). Table 3 lists the most central genes (hubs) in the network with their associated rank and log-likelihood score (Lee et al., 2008). The number of gene connections range from 21 to 1, with the predictive coherence of query genes being 0.896. The predictive coherence is indicated by an area under the receiver operating characteristic curve (AUC) with 0.5 indicating random performance and 1 perfect performance (Lee et al., 2010a). An AUC value of 0.896 indicates a high predictive power, suggesting that the gene interactions and connections displayed are supported by substantial evidence. This predictive power is considerably reduced when the list of differentially expressed genes is partitioned and organized by linkage group, up regulation, down regulation or by JCMS vs. OP50 (data not shown). Thus, we reasoned the best, and more statistically supported approach was to analyze and interpret the genes within one network.

**Table 3:** WormNet v2 network of genes differentially expressed in response to *S. maltophilia* JCMS, K279a or *E. coli* OP50.

**Gene name**	**Sequence name**	**Rank**	**Score**	**C**	**Linked genes**
*pqn-98*	ZK488.7	23	2.05	21	C05E7.3, C12D5.3, C25F9.6, *che-11, acs-17, ftn-1, sue-1*, F07C4.6, F23H11.7, F35A5.2, F55A4.7, *dod-22, lys-5*, H12D21.4, *twk-14, flp-11*, K08D10.10, *xbx-3*, R13H7.2, T05F1.11, and T16G1.4
*cpr-4*	F44C4.3	37	1.76	14	C14C6.2, F07C4.6, *clec-7, lys-10*, F28A12.4, *dod-23, lys-5, lys-6*, F58B4.5, F59D6.3, K01C8.1, R03G5.5, *spp-4*, and T16G1.4
*dod-22*	F55G11.5	2	2.89	13	B0024.4, C02C2.4, *clec-10, mpk-2, ugt-22*, C14C6.2, *pho-1*, F19B2.5, F55G11.8, *clec-67*, K08D8.4, T05F1.11, and *pqn-98*
F28A12.4	F28A12.4	21	2.09	13	*clec-10, ugt-22*, C14C6.2, DH11.2, *cpr-4, lys-6*, F59D6.3, *ugt-62*, R03G5.5, T01D3.6, T05F1.11, *spp-4*, and T22F3.11
*acs-17*	C46F4.2	8	2.56	12	B0391.10, *ugt-51*, C17C3.1, C48B4.1, F02C12.2, F25D1.5, *cwp-4*, R03G5.5, T01G6.10, T12D8.5, *nlp-16*, and *pqn-98*
F19B2.5	F19B2.5	1	2.89	10	B0024.4, *ugt-22*, C25D7.5, F08G2.5, F16H6.10, *dod-22*, F55G11.8, *clec-67*, K08D8.4 and T05F1.11
R03G5.5	R03G5.5	52	1.51	10	*acs-17*, F02C12.2, *clec-7*, F25D1.5, F28A12.4, *cpr-4, lys-6*, F59D6.3, T01G6.10 and T16G1.4
B0024.4	B0024.4	12	2.49	9	C04G6.5, C25D7.5, F08G2.5, F16H6.10, F19B2.5, *dod-22*, F55G11.8, *clec-67* and K08D8.4
*mpk-2*	C04G6.1	24	1.97	9	*ugt-22*, C18H7.4, *rom-2, trk-1, pho-1, clec-28, dod-22*, K11H12.4, and T01D3.6
T22F3.11	T22F3.11	3	2.79	9	*avr-14*, C05E7.3, C14C6.2, F07C4.12, F28A12.4, F46C5.1, *flp-11, asic-2*, and *pqn-97*
*ugt-22*	C08F11.8	30	1.86	9	C02C2.4, *mpk-2, pho-1*, F19B2.5, F28A12.4, *clec-28, dod-22, clec-67*, and T05F1.11
C14C6.2	C14C6.2	35	1.78	8	*clec-10, clec-7*, F28A12.4, *cpr-4, dod-22, clec-67*, T05F1.11, and T22F3.11
C48B4.1	C48B4.1	32	1.83	8	*prx-3*, C27A7.1, *acs-17*, F09F7.4, F14F9.4, F58B4.5, *nhr-144*, and W03F9.4
*clec-67*	F56D6.2	18	2.22	8	B0024.4, *ugt-22*, C14C6.2, F19B2.5, *dod-22*, F55G11.8, K08D8.4, and T05F1.11
F08G2.5	F08G2.5	9	2.56	8	B0024.4, C04G6.5, C05E7.3, C25D7.5, F16H6.10, F19B2.5, F53A9.2, and K08D8.4
*lys-6*	F58B3.3	36	1.78	8	D2023.4, F28A12.4, *cpr-4*, F49C12.14, *dod-23*, R03G5.5, T01D3.6, and *spp-4*
T05F1.11	T05F1.11	55	1.49	8	*ugt-22*, C14C6.2, F19B2.5, F28A12.4, *dod-22, clec-67*, Y37A1B.5, and *pqn-98*
*ftn-1*	C54F6.14	40	1.7	7	*ugt-51*, C27H5.2, DH11.2, F07C4.6, T09B9.3, T12D8.5, and *pqn-98*

*WormNet v2 was queried with all unique significantly differentially expressed genes shown in Table S3. One hundred and eighteen of the three hundred and ninety-three differentially expressed genes had putative connections and are listed by the number of connections in the probabilistic functional gene network model (Figure 3). The WormNet rank and score are listed for each gene. C = Number of genes that are connected to the listed gene. WormNet derived scores are probabilities based on a modified Bayesian integration of likelihood scores from individual datasets. Likelihood score and rank (based on score) are included for each gene. Genes that were predicted to have a functional linkage to the listed gene hub are in the corresponding linked genes column. Only the top 20 most connected hubs are listed here. All genes and/or gene hubs are listed in Table S5*.

### Functional validation of genes central to the network

WormNet inferred interactions have been functionally verified and genes central to a highly connected network have been shown to be more likely to have essential functions (Lee et al., 2008). Thus, we hypothesized that genes central to the identified network (Figure 3) would be fundamental to the *C. elegans* response to pathogenic *S. maltophilia*. Using the data generated from WormNet v2, genes were ranked by their number of predicted interactions (Table S5). We sought loss-of-function alleles of these genes to determine their role in response to different bacterial environments. Fortunately, deletion alleles were available for seven of the top 20 hub genes: *cpr-4* (cysteine protease related), *dod-22* (down-stream of *daf-16*), *acs-17* (fatty acid CoA synthetase), *mpk-2* (mitogen activated protein kinase), *lys-6* (lysozyme), *clec-67* (C-type lectin), and acyl-CoA oxidase C48B4.1. Of these candidates, only knock down of *dod-22* had a documented bacterial pathogen (*V. cholerae*) susceptibility phenotype (Sahu et al., 2012). Thus, for the majority of these genes, the discovery of any bacterial environment related phenotype is novel and aids in our understanding of *C. elegans* innate immunity.

**Figure 4:**
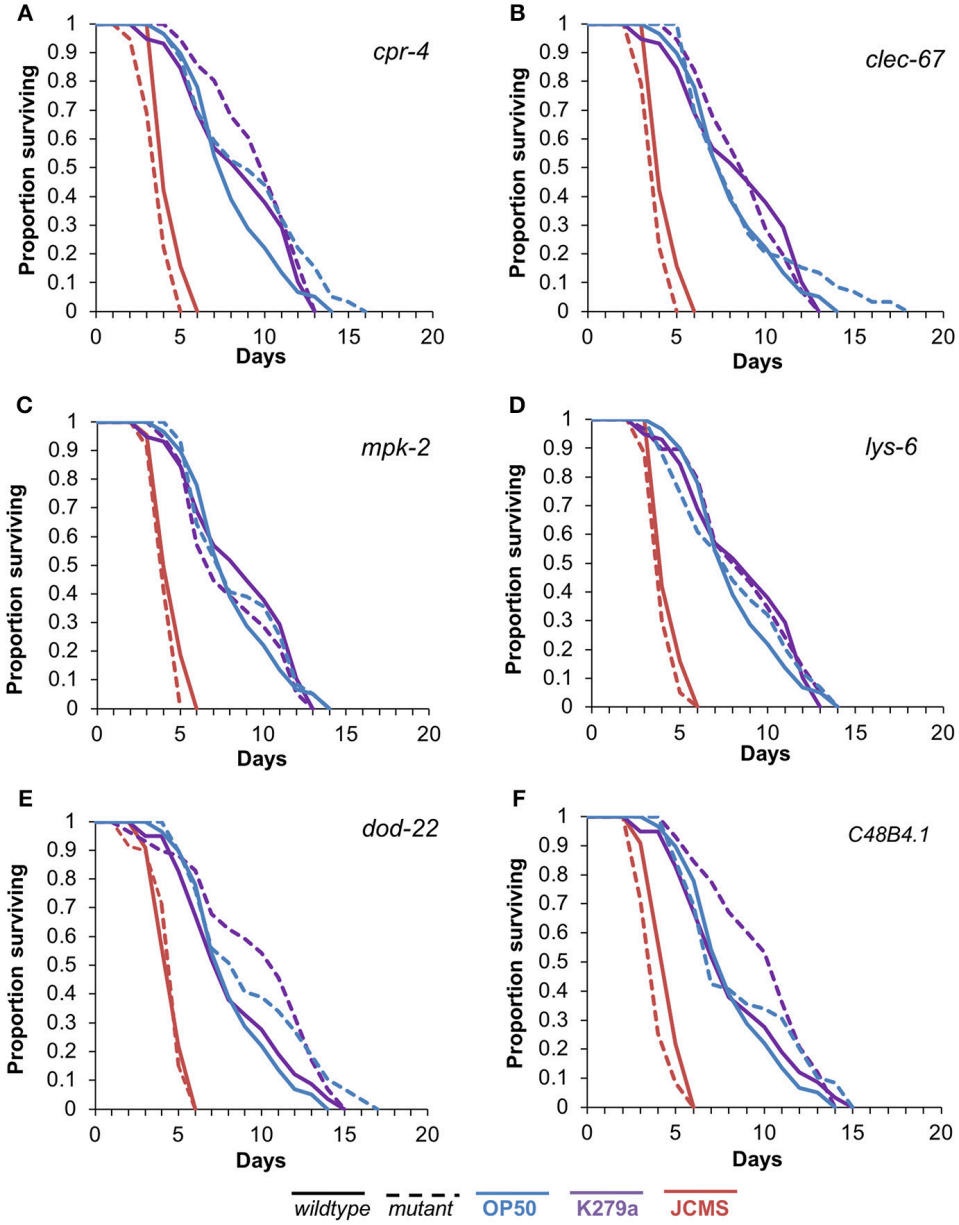
Survival of wild-type nematodes and array candidate mutants on *E. coli* OP50, *S. maltophilia* JCMS or K279a. Survival of wild-type nematodes (solid lines) and select hub gene mutants (dashed lines) exposed to *E. coli* OP50 (blue), *S. maltophilia* JCMS (red) or K279a (purple). **(A)** *cpr-4(ok3413)* **(B)** *clec-67(ok2770)* **(C)** *mpk-2(ok219)* **(D)** *lys-6(ok2075)* **(E)** *dod-22(ok1918)* **(F)** C48B4.1*(ok2619)*. Results plotted are the proportion of surviving worms using Kaplan-Meier estimates for at least three replicate samples (10–15 nematodes per replicate) of the same nematode population. *p*-values from the Cox proportional hazards models and sample sizes of each population are included in Table 4. Mutants of *cpr-4, clec-67, mpk-2*, C48B4.1, and *lys-6* were all significantly susceptible to JCMS, while *dod-22* mutants were resistant to K279a.

To assess the functional role of the selected candidates, we performed survival analysis for each mutant vs. wild-type nematode in response to *S. maltophilia* K279a and JCMS as well as *E. coli* OP50. All of the candidate genes were significantly differentially expressed only between K279a and JCMS. Thus, we hypothesized that mutations in these hub genes would cause a survival phenotype in response to one or both of these bacteria. K279a is also less pathogenic than JCMS and allowed the comparison between avirulent and virulent *S. maltophilia* strains. Mutations in *cpr-4, mpk-2, lys-6, clec-67*, and *C48B4.1* caused hyper-susceptibility to JCMS but did not have a statistically significant survival phenotype on K279a (Figure 4 and Table 4). However, *dod-22* mutants were significantly long lived on K249a. As previously described (White et al., 2016), the degree to which a gene is involved on a given bacterial environment can be inferred from the mutant to *wildtype* Cox proportional hazard ratio (see Materials and Methods). We observed that these mutants that were hyper-susceptible to *S. maltophilia* JCMS had hazard ratios ranging from 2.1 to 1.5 (Table 4). These data indicate that *cpr-4, mpk-2, lys-6, clec-67, dod-22*, and *C48B4.1* have unique roles on one or more of the bacterial environments tested. In summary, six of seven mutants had survivorship phenotypes on JCMS or K279a (Table 4), which validates the network centrality hypothesis.

**Table 4:** Survival of wild-type nematodes vs. gene candidate mutants fed *S. maltophilia* JCMS or K279a.

		***S. maltophilia*** **JCMS**					***S. maltophilia*** **K279a**				
**Genotype**	**FC**	**Reg**.	**M**	**SE**	**N**	**Hazard ratio**	***p*-value**	**M**	**SE**	**N**	**Hazard ratio**	***p*-value**
*wildtype* (WT)	N/A	N/A	4.6	0.105	56	N/A	N/A	8.7	0.4	58	N/A	N/A
*lys-6 (ok2075)*	3.53	Down	4.2	0.092	60	1.455	0.0453	8.8	0.39	58	0.887	0.527
*mpk-2 (ok219)*	1.57a 1.56c	Down	4.3	0.082	58	1.495	0.043	8.1	0.37	56	1.26	0.216
*clec-67 (ok2770)*	2.02	Up	4.0	0.086	58	2.016	4.5E-04	9.0	0.31	56	1.07	0.727
*cpr-4 (ok3413)*	1.60	Down	3.9	0.108	58	2.123	1.6E-04	9.8	0.33	56	0.788	0.204
*acs-17 (ok1562)*	2.23	Down	4.5	0.146	59	1.0	0.962	10	0.29	53	0.738	0.118
*dod-22 (ok1918)*	2.83	Up	4.7	0.134	59	1.02	0.919	10	0.47	59	0.63	0.013
C48B4.1 *(ok2619)*	2.35	Down	4.1	0.116	59	1.75	0.0032	10	0.36	58	0.713	0.073

*Hazard ratios represent the hazard of the treatment divided by the control (wild-type) of the same bacteria. A hazard is the probability that a nematode at a given time dies. p-values are given for the survival predictor of treatment (nematode genotype) for Cox proportional hazard models in R. p < 0.05 were considered significant. Two isoforms (C04G6.1a and C04G6.1c, Table S3) of mpk-2 are differentially expressed. Number of nematodes tested = N. M = mean survival units (days). The fold change (FC) and regulation (Reg.) of each gene is listed to the right of the corresponding genotype*.

We also tested the opposing hypothesis that disconnected differentially expressed genes are less essential and/or functionally relevant and, therefore would be less likely to have a survival phenotype on JCMS or K279a. The genes selected for testing this hypothesis were outside of the WormNet generated network, had deletion alleles available and had expression patterns that were similar to the tested gene candidates (Table S3 and S6). Surprisingly, mutants of four (*tctn-1, kcnl-2, srw-145* and *lgc-11*) of seven genes showed significant survival phenotypes when exposed to both or either of the tested bacterial environments (Figure S2 and Table S6). Mutants of *tctn-1* were significantly susceptible to both JCMS and K279a while, only *lgc-11* or *srw-145* mutants were short lived on JCMS or K279a with hazard ratios ranging from 4.0 to 1.1 (Figure S2 and Table S6). Mutants of *tctn-1* and *srw-145* were also short lived on K279a and were more susceptible to K279a than to JCMS. Lastly, like *dod-22*, loss of *kcnl-2* causes extended survival on K279a. The hazard ratios for *dod-22* and *kcnl-2* mutants on *S. maltophilia* K279a were 0.63 and 0.56, indicating that loss of *kcnl-2* promotes longer life than loss of *dod-22*. Taken together, these data suggest that the information gained from differential expression and network analyses has predictive value for determining gene functions.

## Discussion

In this study we used a transcriptomic approach to identify genes that are involved in the *C. elegans* response to pathogenic (JCMS) and non-pathogenic (K279a) strains of the emerging, universal and opportunistic bacterial pathogen *S. maltophilia* as well as the common *C. elegans* laboratory food, *E. coli* OP50. We observed that 97.2% of the differentially expressed genes were significant for the JCMS vs. K279a comparison (Figure 1). Thus, after 24 h of exposure to these bacteria the transcriptional response is driven by differences in pathogenicity rather than differences driven by bacteria species.

To gain insight into the nematode-bacterial interaction, we performed gene ontology and tissue expression enrichment analysis to determine the functions, processes, cellular components, and tissues that play a significant role in the nematode response. As expected, the GO terms defense response to Gram negative bacteria and/or stimulus, stress, defense, and/or innate immune response were enriched among the upregulated genes (Table 1). Tissue enrichment analysis (Table 2), revealed that these up regulated genes are localized to the intestine, muscular system, striated muscle, AVA and PVD interneurons and/or sensory receptors. The down regulated genes are also associated with the intestine, the amphid, inner labial, and hook sensory receptors, the CEM sensory secretory neuron and the AVE ventral cord interneuron. Thus, it is reasonable to hypothesize that *S. maltophilia* JCMS and K279a elicit different neurological responses that influence nematode behavior in response to these bacteria. Interestingly, *C. elegans* has been shown to avoid pathogenic bacteria and display olfactory learning behavior (Zhang et al., 2005; Pradel et al., 2007), although, this has not yet been shown to be the case for *S. maltophilia*. Furthermore, the intestine is the major digestive and innate immune organ in *C. elegans*, which may explain why most of the differentially expressed genes are localized to this tissue. We observed that the GO terms: component of membrane, transport, oxidation, and reduction and metabolic process were enriched among down regulated genes (Table 1). These terms have been frequently observed in other nematode-bacterial expression studies (Coolon et al., 2009; Irazoqui et al., 2010b); more specifically, anion transport, lipid metabolism and Acyl-CoA dehydrogenases are repressed upon infection with *S. aureus* and *P. aeruginosa* (Irazoqui et al., 2010b).

Although, informative, a substantial number of genes were associated with one or more of the enriched terms, making it difficult to systematically prioritize candidate genes. Furthermore, we sought to discover genes with novel roles in the nematode-bacterial interaction and reasoned that use of gene network analyses might be a more objective approach. We used WormNet v2 (Lee et al., 2010b) to initially reduce our candidate gene list from 393 to 118 genes that had probabilistic connections (Figure 3). To further narrow the list of candidates, we then sorted the genes by the number of predicted connections and the availability of loss of function alleles. Like others (Özgür et al., 2008, 2010), we hypothesized that genes that were central to the network of differentially expressed genes would be required for the response to the bacteria that induced expression changes. This network centrality hypothesis is derived from the finding that proteins, central to a network, evolve more slowly (Hahn and Kern, 2005), which is likely due to pleiotropy (Promislow, 2004), and the increased likelihood that these proteins are involved in an essential process (He and Zhang, 2006). In support of our network centrality hypothesis, six of the seven tested mutants showed differences in survivorship when exposed to JCMS or K279a (Figure 4).

On the other hand, mutations in four of seven genes outside of the network also showed survivorship phenotypes (Figure S2 and Table S6). Except for *tctn-1* (tectonic homolog), these genes, *srw-145* (serpentine receptor, class W), *lgc-11* (ligand-gated ion channel) and *kcnl-2* (potassium K channel-like), were down regulated in response to JCMS vs. K279a (Table S3). Mutants of *tctn-1, lgc-11*, and *srw-145* were susceptible to JCMS and/or K279a; with loss of *tctn-1* being more detrimental on K279a. Intriguingly, *tctn-1* was up regulated on JCMS vs. K279a and mutants showed the highest hazard of all the significant survival phenotypes (Table 4 and Table S6). *lgc-11* only has a role in response to JCMS, while *srw-145* and *kcnl-2* are specifically involved in the response to K279a. None of these genes have any apparent role in nematode innate immunity and/or bacterial response. Yet, all are annotated as an integral component of membrane, which is a significantly enriched GO term. In addition, *kcnl-2* and *lgc-11* are associated with the enriched biological process terms potassium ion transmembrane transport or ion transmembrane transport (Table 1). These genes are also annotated to be expressed in the nervous system that was also shown to be a site of expression by the tissue enrichment analysis (Table 2). Thus, it appears that network centrality is not a sole predictor of gene involvement, and many, if not all, differentially expressed genes likely play some role in the response to *S. maltophilia*.

The *C. elegans* response to the bacterial pathogen *S. maltophilia* JCMS involves the hub genes *lys-6, cpr-4, mpk-2, clec-67*, and *C48B4.1* (Figure 4 and Table 4). None of these genes have a previously documented role in nematode-bacterial interactions. Although, prior studies also showed that C-type lectins (*clec)* and lysozymes (*lys)* played roles in the nematode response to pathogenic bacteria (Irazoqui et al., 2010b; Portal-Celhay et al., 2012), thus it was not surprising to find that *clec-67* and *lys-6* were required for the response to *S. maltophilia* JCMS. In fact, *clec-67* mutants had one of the highest hazards in response to JCMS (Table 4). If lectins are in fact involved in pathogen recognition as postulated (Nicholas and Hodgkin, 2004), this increased hazard may be due to the failure to elicit an immune response. The observation that *clec-67* is not required for response to avirulent K279a and was up regulated on JCMS (Table S3) as well as the human bacterial pathogen *Salmonella enterica* (Kerry et al., 2006) also suggests its invovlement in anti-bacterial defenses. Intriguingly, except for *clec-67*, all the genes were down regulated on JCMS as compared to avirulent K279a (Table S3).

Initially, one might assume that all genes involved in innate immunity would, like *clec-67*, be up regulated in response to a bacterial pathogen. However, it is possible that interaction with the bacterial pathogen leads to down regulation of genes that may be harmful to the bacterium. In support of this conjecture, *P. aeruginosa* infection has been shown to induce the expression of *ins-7* in *C. elegans*, which down regulates genes involved in defense in the intestine (Evans et al., 2008). We propose that this may be the case for many of the hub genes we tested as genes within the same gene families have been shown to be involved in stress and/or innate immune response. For example, the mitogen activated protein kinase (MAPK) gene *mpk-1* is required to combat *M. nematophilum* infection (O'Rourke et al., 2006). Hub gene *mpk-2* also encodes a MAPK and is induced by the bacterial pathogens *S. aureus* and *P. aeruginosa* (Irazoqui et al., 2010b). *C48B4.1*, an ortholog of human acyl-CoA oxidase 1, is implicated in pheromone biosynthesis along with other acyl-CoA oxidases (ACOX-2 and ACOX-3) that are regulated by environmental stressors such as food availability (Zhang et al., 2015). Intriguingly, the lysozyme *lys-6* was needed for *S. maltophilia* resistance but had one of the smallest hazard ratios (Table 4). These data suggest that the destruction of bacteria is needed but is not as imperative as other nematode functions. On the other hand, the cysteine protease encoding gene *cpr-4* has the largest role of the tested hub genes in response to JCMS. *cpr-4* also does not have a demonstrated role in innate immunity. However, protease activity has previously been linked to the nematode-bacterial pathogen response reviewed in Wong et al. (2007) and, *cpr-2*, another cysteine protease encoding gene, is regulated by *S. aureus* infection (Irazoqui et al., 2008). Thus, protease activity, sugar binding and to a lesser extent, fatty acid metabolism and/or oxidation and reduction, MAPK signaling, ion transport/signal transduction and lysozyme activity are all involved in combating *S. maltophilia* JCMS.

The CUB domain containing protein DOD-22 is induced by gram negative pathogens (Alper et al., 2007) and is required for the response to *Vibrio cholerae* (Sahu et al., 2012). We also found that *dod-22* is significantly induced in response to pathogenic JCMS vs. K279a, suggesting that *dod-22* is required for JCMS response. However, *dod-22* mutants are not significantly susceptible to JCMS (Figure 4, Table 4). Thus, the up-regulation of this gene in response to JCMS was not predictive of its involvement. Future studies that include measuring gene expression on JCMS and K279a over the course of nematode survival may reveal a differential expression pattern with greater predictive power. On the other hand, *dod-22* is regulated by *C. elegans* DAF-2/16 signaling (Murphy et al., 2003) and we have demonstrated that this pathway is not involved in the *S. maltophilia* JCMS response (White et al., 2016). Thus, the *dod-22* mutant survival phenotype on JCMS is less surprising and implies that other DAF-2/16 pathway effector genes will also not be involved on these bacteria. Conversely, *dod-22* mutants had significantly extended lifespan in response to K279a which agrees with a role for DAF-2/16 signaling on avirulent bacteria (White et al., 2016).

The data presented here reveal that the nematode innate immune response is more specific to the type of *C. elegans*-bacterial interaction rather than bacteria species or strain. Metabolic regulation and gene products such as mitogen activated protein kinases, cysteine proteases, lysozymes and lectins are necessary for combating bacterial pathogens such as *S. maltophilia*. Therefore, it is reasonable to propose that the nematode response is only strain specific when there are differences in bacterial virulence. These findings will be important to consider in future studies investigating the *C. elegans- S. maltophilia* and other human-bacterial pathogen interactions.

## Author contributions

All experiments except the survival experiments on the non-connected differentially expressed genes were completed by CW under the supervision and guidance of MH. CW also wrote the manuscript and MH provided feedback and edits. MH conducted the TEA and GO analyzes and CW conducted survival, microarray, and quantitative PCR analyzes.

### Conflict of interest statement

The authors declare that the research was conducted in the absence of any commercial or financial relationships that could be construed as a potential conflict of interest.
