# Supplementary material for: Transcriptomic, Functional, and Network Analyses Reveal Novel Genes Involved in the Interaction Between Caenorhabditis elegans and Stenotrophomonas maltophilia
Source: Front Cell Infect Microbiol. 2018 Aug 20;8:266. doi: 10.3389/fcimb.2018.00266 (PMC6109753; doi:10.3389/fcimb.2018.00266)
Supplement: Supplementary file 1 [file Table_1.pdf]

Supplementary Material

Transcriptomic, functional and network analyses reveal novel genes involved in the interaction between *Caenorhabditis elegans* and *Stenotrophomonas maltophilia*

Corin V. White, Michael A. Herman*

*** Correspondence:** Michael A. Herman: mherman5@unl.edu

1.1 Table S1: Primer sequences used for reverse transcription quantitative polymerase chain reaction (RT qPCR).

Reverse transcription quantitative polymerase chain reaction (RT qPCR) was performed on the listed genes for the validation of the observed differential expression patterns. All genes (except the reference gene *csq-1*) were differentially expressed in at least one bacterial treatment comparison and had a minimum fold change of 2.5 (see Microarray analysis in the Materials and Methods section of the manuscript). The cycling conditions for all primer sequences were optimized and we aimed for efficiency between 95% and 100%.

Gene Name(s)	Primer Sequences
csq-1<br>F40E10.3	5’- AACTGAGGTTCTGACCGAGAAG - 3’<br>5’-TACTGGTCAAGCTCTGAGTCGTC - 3’
F53B2.8	5’-GAAGTCGAGAGGAGCAGAAACGAGCC - 3’<br>5’- CGGGGTGGTCTTGGGGCTGG - 3’
W03F9.4	5’- AAACTCTTGTGTCTCTGCTCATC G - 3’<br>5’-CGCTGTCGTTGCATAGCTTGGCTT - 3’
ilys-3<br>C45G7.3	5’-AGCCGCGTGGAAGAGGTGC - 3’<br>5’- TGCATCCTTGTGGCCCTCCG - 3’
F08G2.5	5’- TCTTCCTCGTCCTCTTCTTCCG - 3’<br>5’- ATTGCGGTATGGTTCCCACG - 3’
