# Supplementary material for: Transcriptomic, Functional, and Network Analyses Reveal Novel Genes Involved in the Interaction Between Caenorhabditis elegans and Stenotrophomonas maltophilia
Source: Front Cell Infect Microbiol. 2018 Aug 20;8:266. doi: 10.3389/fcimb.2018.00266 (PMC6109753; doi:10.3389/fcimb.2018.00266)
Supplement: Supplementary file 6 [file Table_6.pdf]

Supplementary Material

Transcriptomic, functional and network analyses reveal novel genes involved in the interaction between *Caenorhabditis elegans* and *Stenotrophomonas maltophilia*

Corin V. White, Michael A. Herman*

*** Correspondence:** Michael A. Herman: mherman5@unl.edu

1.1 Table S6: Survival of wild-type nematodes versus mutants (non-connected differentially expressed genes) on *S. maltophilia* JCMS or K279a.

Four of seven mutants had significant survivorship phenotypes in response to JCMS or K279a. p values are given for the survival predictor of treatment (mutant nematode genotype) for Cox proportional hazard models in R. p values less than 0.05 were considered significant. *Five isoforms (F08A10.1a-e) of *kcnl-2* were differentially expressed (Table S3). Number of nematodes tested = Number. Mean = mean survival units (days). The fold change (FC) and regulation (Reg.) of each gene is listed to the right of the corresponding genotype. Mutants of *lgc-11* and *tctn-1* were short lived on *S. maltophilia* JCMS. *srw-145* and *tctn-1* mutants were susceptible while, *kcnl-2* mutants were marginally significantly long lived on *S. maltophilia* K279a.

			***S. maltophilia* JCMS**					***S. maltophilia* K279a**				
**Genotype**	**FC**	**Reg.**	**M**	**SE**	**N**	**Hazard Ratio**	**p value**	**M**	**SE**	**N**	**Hazard Ratio**	**p value**
wildtype (WT)	N/A	N/A	4.0	0.27	30	N/A	N/A	8.2	0.68	28	N/A	N/A
gcy-14 (pe1102)	2.19	down	5.1	0.18	30	0.63	0.0851	8.2	0.33	29	1.6	0.0894
kcnl-2 (ok2818)	4.40*	down	4.3	0.20	29	0.97	0.913	10.0	0.59	25	0.56	0.0516
numr-1 (ok2239)	2.77	up	4.0	0.15	30	1.2	0.483	9.3	0.49	29	0.77	0.327
srw-145 (ok495)	2.27	down	4.2	0.18	30	1.1	0.825	7.03	0.36	29	2.5	0.0027
acr-7 (tm863)	1.70	down	3.8	0.13	26	1.4	0.206	8.5	0.47	29	1.2	0.486
lgc-11 (tm627)	2.39	down	3.1	0.25	28	1.8	0.0319	10.1	0.38	29	0.66	0.128
tctn-1 (ok3021)	2.0	up	2.8	0.25	30	4.0	1.9E-06	4.0	0.36	17	6.1	2.8E-06
