# Supplementary material for: Transcriptomic, Functional, and Network Analyses Reveal Novel Genes Involved in the Interaction Between Caenorhabditis elegans and Stenotrophomonas maltophilia
Source: Front Cell Infect Microbiol. 2018 Aug 20;8:266. doi: 10.3389/fcimb.2018.00266 (PMC6109753; doi:10.3389/fcimb.2018.00266)
Supplement: Supplementary file 7 [file Image_1.pdf]

Supplementary Material

Transcriptomic, functional and network analyses reveal novel genes involved in the interaction between *Caenorhabditis elegans* and *Stenotrophomonas maltophilia*

Corin V. White, Michael A. Herman*

*** Correspondence:** Michael A. Herman: mherman5@unl.edu

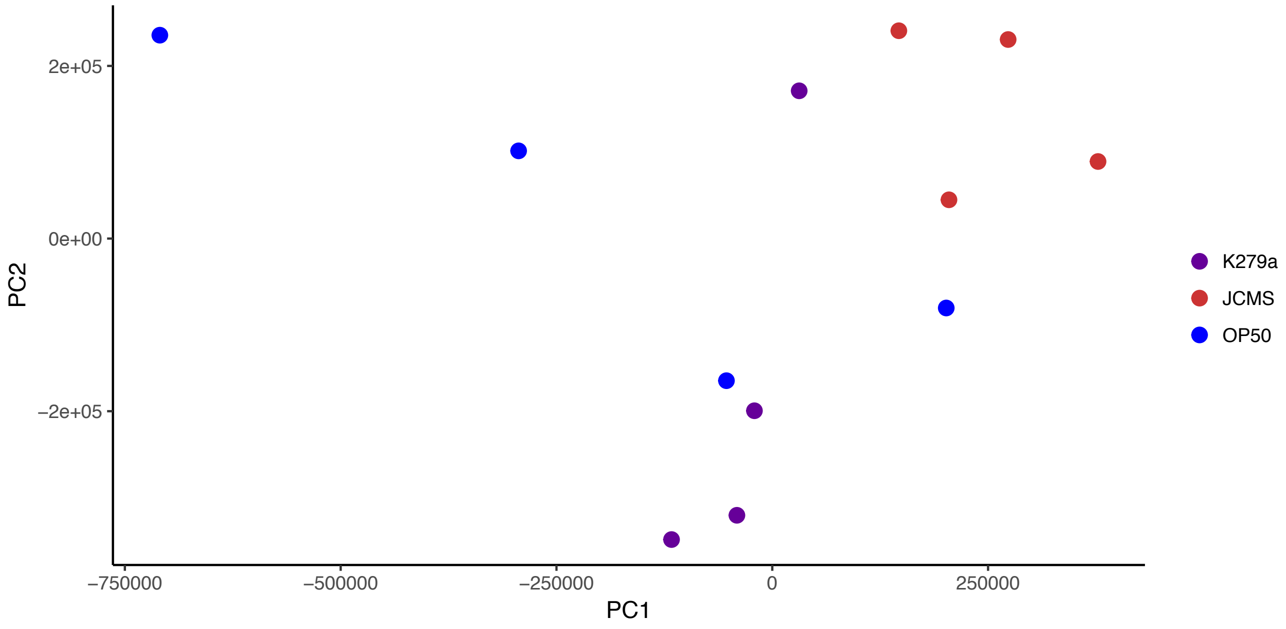

Figure S1: Principal component analysis of expression profile biological replicates

Singular value decomposition was used to construct a principle component analysis using the function ggplot in R within the ggplot2 package. PCA was used along with correlation coefficients to evaluate the similarity between four biological replicates for each bacterial treatment. Each point on the PCA represents the normalized expression data on K279a (purple), JCMS (red) and OP50 (blue). Replicates for treatment with JCMS and K279a are more tightly clustered while replicates for OP50 are loosely related. One replicate (556640A12) for OP50 was not included in downstream analysis because this sample had the lowest correlation coefficients (Table S2) and is the most un-clustered (upper left hand corner) sample on this PCA plot. The variance explained for principal component 1 is 32.27% and the variance explained for principal component 2 is 17.83%.
