# Supplementary material for: Transcriptomic, Functional, and Network Analyses Reveal Novel Genes Involved in the Interaction Between Caenorhabditis elegans and Stenotrophomonas maltophilia
Source: Front Cell Infect Microbiol. 2018 Aug 20;8:266. doi: 10.3389/fcimb.2018.00266 (PMC6109753; doi:10.3389/fcimb.2018.00266)
Supplement: Supplementary file 8 [file Image_2.pdf]

frontiers

Supplementary Material

Transcriptomic, functional and network analyses reveal novel genes involved in the interaction between *Caenorhabditis elegans* and *Stenotrophomonas maltophilia*

Corin V. White, Michael A. Herman*

*** Correspondence:** Michael A. Herman: mherman5@unl.edu

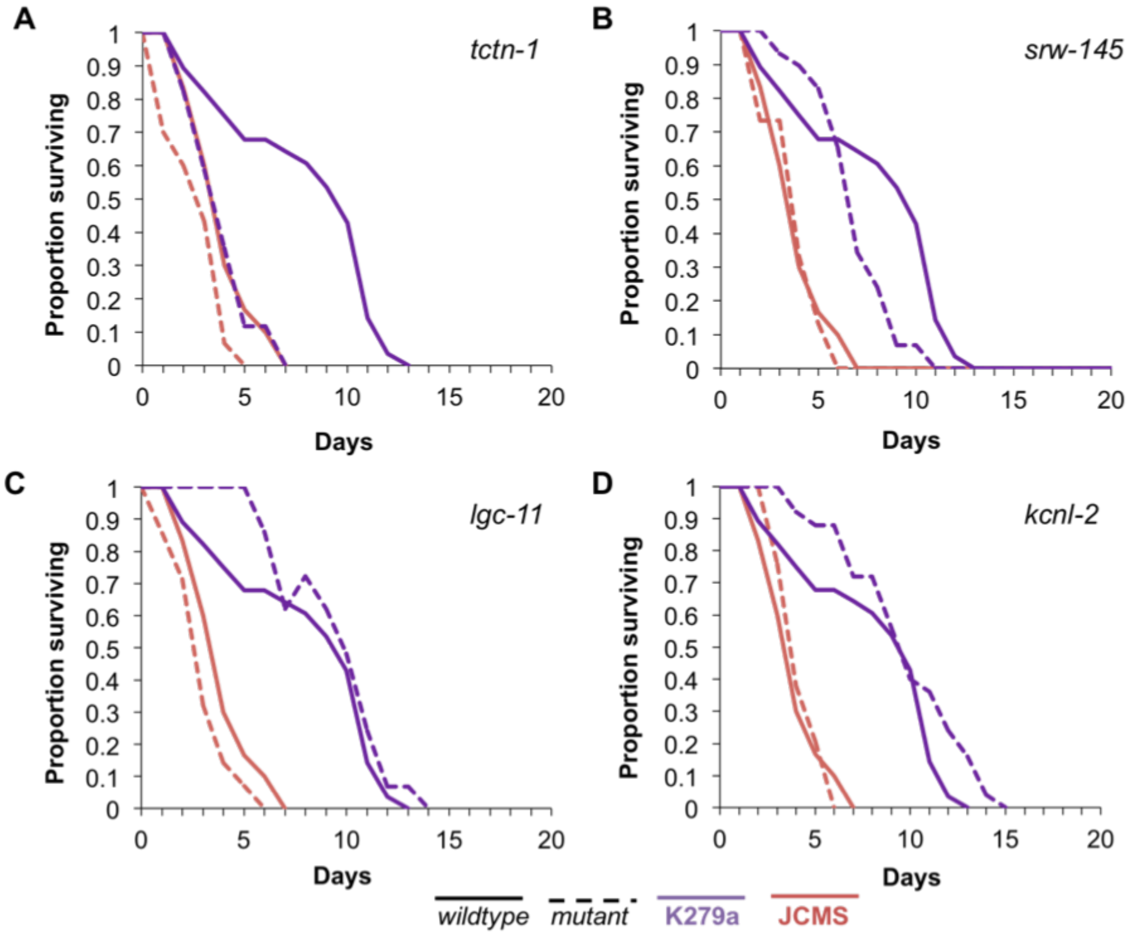

Figure S2: Survival of wild-type nematodes and mutants (non-connected differentially expressed genes) on *S. maltophilia* JCMS or K279a.

Survival of wild-type nematodes (solid lines) and select mutants in genes with similar expression representative patterns that are not part of the gene network (dashed lines) exposed to *S. maltophilia* JCMS (red) or K279a (purple). **(A)** *tctn-1(ok3021)* **(B)** *srw-145(ok495)* **(C)** *lgc-11 (tm627)* **(D)** *kcnl-2(ok2818)*. Gene mutants shown were those that showed significant changes in survival on JCMS or K279a (Table S6). Results plotted are the proportion of surviving worms using Kaplan-Meier

estimates for replicate samples (starting with 10 nematodes per replicate) of the same nematode population. p values from the Cox proportional hazards models and sample sizes of each population are included in Table S6. Mutants of *lgc-11* and *tctn-1* were short lived on *S. maltophilia* JCMS. *srw-145* and *tctn-1* mutants were susceptible while, *kcnl-2* mutants were marginally significantly long lived on *S. maltophilia* K279a. Survival on *E. coli* OP50 was not conducted for all mutants and, thus, these data were not included in this figure.
